# Mastering your fellowship: Part 1, 2025

**DOI:** 10.4102/safp.v67i1.6024

**Published:** 2025-01-13

**Authors:** Klaus B. von Pressentin, John M. Musonda, Mergan Naidoo, Michéle Torlutter, Gert Marincowitz, Selvandran Rangiah, Zakariya Badat

**Affiliations:** 1 Division of Family Medicine, Department of Family, Community and Emergency Care, Faculty of Health Sciences, University of Cape Town, Cape Town, South Africa; 2 Department of Family Medicine and Primary Care, Faculty of Health Sciences, University of the Witwatersrand, Johannesburg, South Africa; 3 Department of Family Medicine, College of Health Sciences, University of KwaZulu-Natal, Durban, South Africa; 4 Department of Family Medicine, University of Limpopo, Polokwane, South Africa

**Keywords:** family physicians, FCFP (SA) examination, family medicine registrars, postgraduate training, national exit examination, child health

## Abstract

The series ‘Mastering your Fellowship’ provides examples of the question formats encountered in the written and clinical examinations, Part A of the Fellowship of the College of Family Physicians of South Africa (FCFP [SA]) examination. The series aims to help family medicine registrars (and supervisors) prepare for this examination.

## Introduction

This *South African Family Practice* journal section aims to help registrars prepare for the Fellowship of the College of Family Physicians of South Africa (FCFP [SA]) Final Part A examination. It provides examples of the question formats encountered in the written exam: multiple choice question (MCQ) in the form of single best answer (SBA – Type A) and extended matching question (EMQ – Type R); short answer question (SAQ), questions based on the critical reading of a journal article (CRJ: evidence-based medicine) and an example of an objectively structured clinical examination (OSCE) question. Each of these question types is presented based on the College of Family Physicians blueprint and the key learning outcomes of the FCFP (SA) programme. The MCQs draw on the 10 clinical domains of family medicine, the SAQs align with the five national unit standards, and the critical reading section includes evidence-based medicine and primary care research methods.

This edition aligns with all five unit standards described in Mash R, Steinberg H, Naidoo M. Updated programmatic learning outcomes for the training of family physicians in South Africa. *S Afr Fam Pract.* 2021;63(3):a5342. https://doi.org/10.4102/safp.v63i1.5342. The clinical domain covered in this edition is child health. We suggest you attempt to answer the questions (by yourself or with peers and supervisors) before finding the model answers online: http://www.safpj.co.za/.

Please visit the Colleges of Medicine website for guidelines on the Fellowship examination: https://cmsa.co.za/fellowship-of-the-college-of-family-physicians-of-south-africa-fcfpsa/.

We are keen to hear how this series assists registrars and their supervisors in preparing for the FCFP (SA) examination. Please email us (editor@safpj.co.za) with your feedback and suggestions.

## Extended matching question (EMQ)

### Theme: An approach to the child who presents with a painful hip

#### Options

- Developmental hip dysplasia.
- Growing pains.
- Osgood–Schlatter’s disease.
- Osteomyelitis of the hip.
- Perthes’ disease.
- Septic arthritis.
- Slipped upper femoral epiphysis.
- Transient synovitis.

#### Instructions

For each clinical scenario discussed further in the text, choose the most appropriate option from the options above. Each answer may be used once, more than once or not at all.

#### Scenarios

1. An 8-year-old boy is accompanied by his mother to the clinic. He complains of pain in his left hip joint, which developed after participating in school sports. The hip is tender on weight-bearing, and you notice a limp when walking. The pain resolves within 2 weeks.
2. A 12-year-old girl with a body mass index of 29 kg/m^2^ presents to the clinic complaining of left thigh and hip pains for 2 weeks. She has a slight limp, limited abduction and medial rotation of the hip joint movement.

Short answer:

**Scenario 1:** h. Transient synovitis

**Scenario 2:** g. Slipped upper femoral epiphysis

### Discussion

A limping child with hip pain is a common diagnostic challenge. Understanding the common causes of hip pain in children is essential for accurate diagnosis and treatment. A comprehensive assessment ensures appropriate patient management.

- *Developmental hip dysplasia*: Occurs when the acetabulum (hip socket) is too shallow to firmly hold the femoral head, potentially affecting one or both hip joints at any age from birth onwards. A limp may be present when the child starts walking.
- *Growing pains*: These typically affect children under 10 years and present as pain in the hips, knees and other joints. They may wake the child at night and be relieved by massaging the affected joint. The child usually settles back to sleep within an hour and is otherwise well.
- *Osgood–Schlatter’s disease*: A tibial tubercle apophyseal traction injury following sports activities, particularly running and jumping. It is common in athletic teenagers, who may present with pain, tenderness and swelling over the tibial tubercle.
- *Osteomyelitis of the hip*: An infection of the bone, bone marrow or bone tissue, which may present with hip pain, reduced weight, fever and rigours. The disease may affect the hip joint, particularly in older children (metaphysis) and children under 18 months (epiphysis).
- *Perthes’ disease*: Hip osteonecrosis that develops over a month and affects 4–7 year-olds. Patients may present with hip pain, a consistent limp and a limited range of hip joint movement. The cause is the disruption of blood flow, leading to aseptic necrosis of the femoral head.
- *Septic arthritis*: A surgical emergency, as infection affects highly vascularised intra-articular joints like the hips, knees and shoulders, potentially causing destructive arthropathy. It commonly affects children under five and presents with fever, general malaise and avoidance of hip movements.
- *Slipped upper femoral epiphysis*: This is common in obese children aged 10–15 years, who may have delayed skeletal maturation. The upper femoral epiphysis slips to the femur in the posteroinferior position, presenting with a limp and limited hip movement, particularly abduction and medial rotation. The affected leg may be externally rotated and shortened.
- *Transient synovitis*: This condition typically occurs between ages 3 years and 10 years and presents with transient hip pain, a slight limp, limited hip movement or immobilisation of the child. The exact cause is unknown, but the condition is self-limiting, with symptoms lasting 5–14 days. There is often a history of preceding upper respiratory tract infection or mild trauma, about 2–3 weeks before symptoms appear. It is the most common cause of a limp in children.

**Further reading**

- Mushtaq N, Osmani H, Patel J, Alwan S, Sarraf K, Ahmed N. Evaluation of paediatric hip pain. Br J Hosp Med. 2023;84(7):1–10. https://doi.org/10.12968/hmed.2023.0054
- Price J, Heinz P. A practical approach to joint pain in children. Paediatr Child Health. 2022;32(2):43–49. https://doi.org/10.1016/j.paed.2021.11.001
- Yagdiran A, Zarghooni K, Semler JO, Eysel P. Hip pain in children. Dtsch Ärztebl Int. 2020;117(5):72–82. https://doi.org/10.3238/arztebl.2020.0072

## Short answer question (SAQ): The family physician’s role as a capacity builder and consultant

*This question was previously used in a written FCFP (SA) paper.*

You are the family physician in a district hospital. The facility manager shares her concern with you that the facility is struggling to meet several national targets concerning childhood conditions, especially following the coronavirus disease 2019 (COVID-19) pandemic. Immunisation rates are below target, and conditions such as pneumonia, diarrhoea and severe acute malnutrition in the under-5 year age groups have been increasing in the facility over the last 3 years, along with increased deaths from these conditions.

You supervise a team of medical officers (MOs), community service doctors, interns and family medicine registrars in the facility. The district hospital is the referral centre for primary care facilities in the surrounding sub-districts:

1. In your role as a capacity builder, list four ways in which you might analyse the training needs of the clinicians and primary health care (PHC) providers to address the challenges with child health. Give an example of each. (4 marks)
2. You are on call when a junior MO calls you in a panic from the emergency centre to assist with resuscitating a 1-year-old baby who presents with severe dehydration. She had tried in vain to establish intravenous access, and the baby was rapidly deteriorating. The nurse had suggested an intraosseous infusion, but the junior MO felt unsure of the procedure as she had never done one before. You immediately respond and assist the MO with the resuscitation.
  Following this experience, use Kolb’s learning cycle framework to describe how you can assist this MO with their future professional growth. (6 marks)
3. You have determined a gap in skills and would like to ensure that all the junior doctors in the facility are able to resuscitate children. How would you implement a training session for this? (6 marks)
4. List four ways in which you could carry out your duties as a consultant to the medical team in the facility to improve clinical care for children. (4 marks)
5. How would you know that your efforts to improve capacity to manage sick children in this facility have worked? (5 marks)

**Total: 25 marks**

*Suggested answers (the answers should show some application to the scenario)*

1. In your role as a capacity builder, list four ways in which you might analyse the training needs of the clinicians and PHC providers to address the challenges with child health. Give an example of each. (4 marks)

Model answer: Four relevant points from the list below:

- Audit clinical care to identify areas of poor quality. Perform a root cause analysis, process mapping or operational analysis concerning the management of children in the facility.
- Survey to see prior training in the district hospital and PHC facilities. That is, determine the percentage of health workers trained in Integrated Management of Childhood Illness (IMCI), neonatal resuscitation, Emergency Triage Assessment and Treatment (ETAT) course, Paediatric Advanced Life Support (PALS) course or doctors having completed the Diploma in Child Health (DCH).
- Establish gaps in training. This may be done by surveying the potential audience’s learning needs. Identify clinicians with the potential to train others. Build a team of trainers through insourced or outsourced support or outreach visits.
- Identify problem areas from morbidity and mortality (M&M) meetings.

2. You are on call when a junior MO calls you in a panic from the emergency centre to assist with resuscitating a 1-year-old baby who presents with severe dehydration. She had tried in vain to establish intravenous access, and the baby was rapidly deteriorating. The nurse had suggested an intraosseous infusion, but the junior MO felt unsure of the procedure as she had never done one before. You immediately respond and assist the MO with the resuscitation. Following this experience, use Kolb’s learning cycle framework to describe how you can assist this MO with their professional growth.

Use Kolb’s learning cycle to structure how you would mentor this colleague and facilitate a journey of personal and professional growth and development (*half mark for the heading and 1 mark for the description*) (6 marks in total).

- *Feeling* (*concrete experience*): This is a simple recall of what happened during the resuscitation without reflecting on the correct, incorrect or deficient actions. It answers the question, ‘What happened?’
- *Looking* (*reflective observation*): Discuss the experience with the MO, help them observe and reflect on what happened and provide feedback on what they did and the optimal approach to this emergency. This reflection can be facilitated by using resources such as guidelines and algorithms.
- *Thinking* (*abstract conceptualisation*): This requires an action from you as the mentor, such as assisting (encouraging) the MO to identify what they have learnt in more abstract terms and make a mental shift in how they will approach this type of emergency in the future. This emerges from the reflection using the learning resources, which helps shape the MO’s thinking.
- *Doing* (*active experimentation*): This requires action from you as the mentor. That is, guide the MO on how best to implement the new competencies, including intraosseous access and infusion, into their practice, with support as needed while they gain confidence. The supervisor could possibly arrange a simulated training event for intraosseous (IO) insertion to facilitate the development of confidence.

3. You have determined a gap in skills and would like to ensure that all the junior doctors in the facility are able to resuscitate children. How would you implement a training session for this?

*Describe three ‘what’, ‘how’ and ‘so what’ in depth for 2 marks each (the ‘why’ is in the stem above and implied in the question).* (Total: 6 marks)

- *What*: Establish *clear aims and learning outcomes* for your educational activity (knowledge, skills and attitudes) based on who your participants are. (1 mark)
  ■ Create a content outline, review existing materials, organise and develop new content and decide on the format. Relevant content sources may include IMCI and resuscitation guidelines for neonates and children. (1 mark)
- *How*: Plan your *teaching method* – that is, how the learners will engage with the material, each other, and the tutor as part of the learning experience. Continuous professional development (CPD) meetings, small group learning, clinical training sessions/skills centre, bedside clinical demonstrations, workshops, outreach. (1 mark)
  ■ Manage the *logistics – venue, equipment, time, refreshments and CPD accreditation*. (1 mark)
- *So what*: *Evaluate* your activity and decide how you will know if it has been effective and how you will obtain feedback on how the learners found the learning experience. (1 mark)
  ■ *Revise* your teaching and learning activity based on the feedback and your own experience of the process. (1 mark).

4. List four ways in which you could carry out your duties as a consultant to the medical team in the facility to improve clinical care for children.

*Any four relevant points for 1 mark each (total of 4 marks).* The family physician performs consultant duties in the facility by:

- The family physician works alongside colleagues in child health, providing role modelling for the doctor–patient interaction.
- The family physician should be available for clinical advice – on-site, telephonically or electronically.
- The family physician should assess the competence of new and old staff. What is the previous training of new staff, and how do they perform in observed consultations, case presentations, skills performance and attitudes?
- The family physician may regularly conduct teaching activities (such as ward rounds) in the paediatric ward.
- The family physician performs clinical audits, reviews the findings with the clinical team and co-creates interventions where needed.

5. How would you know that your efforts to improve capacity to manage ill children in this facility have worked?

Has there been a change in clinical practice? Any five relevant applied points: (5 marks)

- The implementation of guidelines has taken place (chart review on paediatric admissions in the hospital, guidelines followed, IMCI implemented in the clinics).
- Assess knowledge gained – that is, quiz.
- Assess and observe practice (skills and attitudes) – for example, direct observation of procedural skills (DOPS).
- Audit on availability of equipment and drugs – for example, intubation equipment and drugs for neonates and children, intraosseous needles and nasogastric tubes.
- Audit appropriate and timeous referrals to regional facilities and quality of care.
- Appropriate health information on child health shared with patients and the community on childhood health and illnesses.
- Morbidity and mortality reviews.
- Improved child health indicators.
- Survey health worker attitudes and professionalism and perceptions of paediatric care.
- Patient and staff satisfaction surveys.
- Staff professional behaviour concerning their peers and patients.

**Further reading**

- Chapters 183 (how to plan and implement a teaching activity), 184 (how to have effective learning encounters in the workplace), 185 (how to facilitate small-group learning) and 187 (how to mentor a colleague). In: Mash B, Brits H, Naidoo M, Ras T, editors. SA family practice manual. Cape Town: Van Schaik, 2023; p. 735–746; 752–755.
- Chapter 9: Developing the primary care team. Mash B, editor. Handbook of family medicine. 4th edition. Oxford University Press Southern Africa; 2017.

## Critical appraisal of quantitative research

*This question was previously used in a written FCFP (SA) paper.*

Read the accompanying article carefully and answer the following questions. As far as possible, use your own words. Do not copy out chunks from the article. Be guided by the allocation of marks concerning the length of your responses.

- Gomwe H, Seekoe E, Lyoka P, Marange CS, Mafa D. Physical activity and sedentary behaviour of primary school learners in the Eastern Cape province of South Africa. S Afr Fam Pract. 2022;64(2):a5381. https://doi.org/10.4102/safp.v64i1.5381

**Total: 30 marks**

1. Comment on the title of the article regarding the PICO (population, interest, context, no outcome) questions. (4 marks)
2. Motivate if the study design applies to the aim of the research. (2 marks)
3. Critically appraise the sampling method used in this research. (5 marks)
4. Comment on the validity of the data-collection instruments. (5 marks)
5. Comment on whether the authors discussed all the relevant limitations of the study. (3 marks)
6. Critically analyse how the authors presented the sedentary behaviour results. (2 marks)
7. Critically appraise the discussion section of the article. (5 marks)
8. Share and justify your reaction (last ‘R’ in READER [R - relevance; E - education; A - applicability; D - discrimination; E - evaluation; R - reaction]) to this article. (4 marks)

*Suggested answers:*

1. Comment on the title of the article regarding the PICO questions. (4 marks)

The title includes the participants (primary school learners), the area of interest (physical activity and sedentary behaviour) and the context (primary schools in the Eastern Cape [EC] province of South Africa) where the research was conducted. This study has no intervention, comparison or outcome. The outcome can also be interpreted as what the study achieved, and in this study, it was to evaluate or describe. Although not written, the title implies that it is a description or evaluation (as stated in the aim):

- P – Population: The population in this title is clear – primary school learners. (1 mark)
- I – Interest: The phenomenon of interest relates to a defined activity, process or event (in the case of this paper, it is physical activity and sedentary behaviour). (1 mark)
- C – Context: The context is the setting or distinct characteristics (primary schools in the EC). (1 mark)
- O – No outcome; the study is descriptive in its design. (1 mark)

2. Motivate if the study design applies to the aim of the research. (2 marks)

- This is a cross-sectional observational study design that collects and describes data of variables collected at one given point in time across a sample population.
- This design was applicable as the study aimed to evaluate (or describe) the area of interest (to measure physical activity and sedentary behaviour) in a defined population (primary school learners in the EC).

3. Critically appraise the sampling method used in this research. (5 marks)

(Any five of these points)

- The section ‘Study setting, sampling and sample size determination’ describes all three topics mixed in one paragraph with little distinctions between the areas; this unstructured approach makes it difficult to read and understand these distinct components of the methods section. (1 mark)
- The authors do not present any sample size calculation. They assume that 18 schools with 10% (a 10th) of children meeting the inclusion criteria selected will be sufficient. This may or may not be sufficient to reach the representativity of the study population, making the study potentially underpowered. (1 mark)
- The districts were chosen ‘conveniently and purposively’, a non-random or non-probability sampling method that limits generalisability to the entire EC population. The districts’ schools were chosen randomly from within specific quintiles, and the children within the schools were chosen randomly. The schools’ inclusion criteria (quintile 1–3) also limit generalisability to the entire EC learner population. (1 mark)
- Stratified random sampling (stratification is by municipalities, then quintiles, then schools and then children: two districts and one metro out of six districts and two metros) was used to select schools randomly; however, the three municipalities were selected both ‘conveniently and purposively’ to represent the ‘diverse contexts’ in the province. Participants were selected randomly in the schools. (1 mark)
- They define the total population of primary school children for the province but not for the three districts in the survey – and the population seems limited to 9- to 13-year-olds. It is unclear what the population size for these three districts is, which is needed for the sample size calculation. (1 mark)
- Likewise, the rationale for choosing 18 out of 5589 EC schools is not explained. (1 mark)
- The final sample has 40% boys and 60% girls – the question is then: does this reflect the actual demographics of the population, or could there be some bias? The sample of learners was selected randomly, so there should not be a bias. However, it is unclear if the study population has a similar distribution of genders. (1 mark)

4. Comment on the validity of the data-collection instruments. (5 marks)

- The researchers used the Physical Activity Questionnaire for Older Children (PAQ-C), previously validated in an ethnically diverse and similar cohort of South African children. (1 mark)
- Sedentary behaviour was measured by a modified self-administered physical activity checklist (SAPAC). The authors decided on a 2-h cut-off value based on the American guidance (American Academy of Paediatrics). There is no evidence if they validate this checklist for our setting and if the 2-h value applies. (1 mark)
- They refer to studies where the SAPAC tool was validated (reference 37) but do not provide information on the contexts of these validation studies. (*This is not part of the model answer; reference 37 cites a study conducted in New Orleans, United States*). (1 mark)
- They also described that they conducted a pilot study to test their understanding of the instrument and mentioned how they adjusted some terms unfamiliar to South African settings (e.g., cross-country skiing, ice hockey, and badminton). (1 mark)
- The authors do not state in what language the PAQ-C was administered, nor do they detail the process (if any) of checking the accuracy of translations. (1 mark)

5. Comment on whether the authors discussed all the relevant limitations of the study. (3 marks)

(Any 3 of these points)

- The authors only discuss one limitation: participants could have forgotten what they did in the past 7 days (recall bias). They argue that although it could have introduced an error, the study established a general idea of the time spent on physical and sedentary activities. (1 mark)
- Other areas that could be limitations or could have introduced bias:
  - Language (there is no reference to the language). Where the questionnaires are available in English or translated to vernacular). (1 mark)
  - The reading and writing abilities of learners. What happened to learners without adequate reading and writing skills? How did learners without adequate reading and writing skills fill out the questionnaire? Were they assisted? It is a concern in South Africa and certainly in the EC that many learners cannot read with understanding. (1 mark)
  - It was unclear from the article if the questionnaire was self-administered versus research assistant administered. (1 mark)
  - Potentially, the purposive sampling of municipalities also introduced selection bias, especially if there is an element of convenience in choosing the municipalities. (1 mark)
  - The results are generalisable only to the primary school children in the specific quintiles in the study population and not all the children in the EC. (1 mark)

6. Critically analyse how the authors presented the sedentary behaviour results. (2 marks)

(Any 2 of the below)

- The authors list the most prevalent sedentary activities and present all in Figure 1 (in the article) diagrammatically. (1 mark)
- The authors also comment on the differences in these activities between gender groups. In fact, the gender differences in specific sedentary behaviours were not analysed for statistical differences. (1 mark)
- The figure makes it difficult to see the underlying results, including the gender differences. It would be better if the results were presented in a table where the reader can see the results and statistical differences. (1 mark)
- The text refers to the gender difference being ‘negligible’, but provides no statistical evidence for this. (1 mark)

7. Critically appraise the discussion section of the article. (5 marks)

*Critical appraisal of the discussion*: (*Any 5 of the below*)

- Much of the text is spent re-iterating the findings and doesn’t consider reasons for the findings. (1 mark)
- The findings seem to contradict those of other researchers in some ways, yet no explanation is given for these differences. The different findings are simply stated as contradictions. The authors should consider whether the differences might be because of methods, context, timeframe or other reasons. (1 mark)
- They consider some underlying reasons for their findings but do not explore them sufficiently:
  - Boys like outdoor competitive games more than girls, and girls prefer indoor activities and household chores. The evidence for this strong statement must be presented, and the gender issues must be unpacked. How do families socialise their children in this way? (1 mark)
  - Rural students have longer walks and more physical chores – this seems to be a reasonable explanation and should be supported by evidence. (1 mark)
  - Urban students have easier access to transport yet are more worried about crime and violence. (1 mark)
  - Leading a healthy lifestyle is too expensive and is not really explained or justified as a serious explanation. (1 mark)
  - Loss of Physical Education in curricula for Life Orientation – this seems like an important consideration. Does this explain the difference between ages? (1 mark).

2. Share and justify your reaction (last ‘R’ in READER) to this article. (4 marks)

To develop a ‘reaction’ to the article, all the previous steps of the READER review must have been considered: (1 mark)

***Explanation of the other steps for clarity but not for marks*:**

- *For a family physician in South Africa, the topic of physical activity in children is very relevant.*
- *The study might not be that educational because there is a general concern about increased sedentary behaviour among children. It is good to see it proven in a methodologically relatively sound study, though there are concerns about generalisability.*
- *The study applies to generalists who work in public health (areas with quintile 1–3 schools).*
- *The study has a reasonably sound methodology: an adequate sample and differences statistically motivated.*
- *The study’s evaluation is as follows: It is a sound study that adequately describes the prevalence of physical activity and sedentary behaviour in the target population.*

Coming to REACTION:

1. The article can be kept for further consideration. (1 mark)
2. Motivation: Considering the generalisability limitations, the very relevant and methodologically relatively sound study strongly argues that primary school children are not active enough in the EC’s specific setting. The sampling makes the results not generalisable to all primary school children and, therefore, not applicable to all settings. (1 mark)
3. Per se, the findings call for action from those involved with children and those who see their health as suggested in the recommendations. Unfortunately, no specific interventions were suggested but referenced. There are no recommendations on how to implement changes in family practice. (1 mark)

*(Any other reactions that are well motivated to be considered).*

**Further reading**

- Pather M. Evidence-based family medicine. In: Mash B, editor. Handbook of family medicine. 4th ed. Cape Town: Oxford University Press, 2017; p. 430–453.
- MacAuley D. READER: an acronym to aid critical reading by general practitioners. Br J Gen Pract. 1994;44(379):83–85.
- The Critical Appraisals Skills Programme (CASP). CASP checklists [homepage on the Internet]. 2023 [cited 2023 Dec 12]. Available from: https://casp-uk.net/casp-tools-checklists/

## Objectively structured clinical examination (OSCE) station scenario: Child health

*Objective*: This station tests the candidate’s ability to advise and assist an intern in managing a child presenting with Hypoxic Ischaemic Encephalopathy (HIE).

*Type of station*: Integrated consultation.

*Role players*: Intern and a neonate mannikin.

### Instructions for candidates

- You are the family physician working at the consultant clinic of a large district hospital. An intern calls you to assist him in managing a neonate who was born 3 days ago and is currently having uncontrolled seizures.
- Your task:
  ■ Assist and supervise the intern in managing this child.
  ■ Establish the diagnosis and determine severity and prognosis.

### Instructions for the examiner

- This is an integrated consultation station in which the candidate has 20 min.
- Familiarise yourself with the assessor guidelines, which detail the expected responses from the candidate.
- No marks are allocated. In the mark sheet (Table 1), tick off one of the three responses for each competency listed. Ensure you are clear on the criteria for judging a candidate’s competence in each area.

**TABLE 1:** Marking sheet for consultation station.

Competencies	Candidate’s rating		
	Not competent	Competent	Good
Establishes and maintains a good doctor–intern–patient relationship	-	-	-
Gathering information: history, examination and investigations	-	-	-
Clinical reasoning	-	-	-
Explanation and planning	-	-	-
Management	-	-	-

### Guidance for examiners

- The aim is to establish that the candidate has an effective and safe approach to managing a neonate presenting with severe HIE, a severe reperfusion injury 72 h post initial insult.
- In addition, the candidate is expected to supervise an intern on the above.
- A working definition of competent performance is when the candidate effectively completes the task within the allotted time in a manner that maintains patient safety, even though the execution may not be efficient and well-structured:
  ■ *Not competent*: patient safety is compromised (including ethically and legally), or the task is not completed.
  ■ *Competent*: the task is completed safely and effectively.
  ■ *Good*: in addition to displaying competence, the task is completed efficiently and in an empathic, patient-centred manner (acknowledges patient’s ideas, beliefs, expectations and concerns/fears).

Establishes and maintains a good clinician–intern relationship:

- The competent candidate respectfully engages with the intern in a dignified manner.
- (*Ascertains reason for the consultation and makes the intern feel comfortable while ensuring that patient safety is maintained).*
- The good candidate *institutes emergency management by assisting the intern in aborting the seizures and explaining the choice of medications that can be used.*

Gathering information:

- The competent candidate gathers sufficient information to establish a clinical assessment. (*Child born 3 days ago – normal vaginal delivery, Apgar score 5/10 and 6/10, prolonged and difficult labour, child required resuscitation at delivery, mother primigravida – 18 years old. The mother was booked and was at term, with no complications during pregnancy.)*
- The good candidate asks about management since birth, that is, in the last 3 days, such as whether the *child was kept hypothermic, arterial blood gas at birth – respiratory acidosis, urine dipsticks for proteins – 1+, full blood count – normal, and cranial ultrasound – not done.*

### Clinical reasoning

The competent candidate determines the HIE score *(Level of consciousness – comatose* [3]; *tone – flaccid* [3]; *seizures – frequent* [2]; *posture – strong flexion* [2]; *Moro, grasp, suck reflexes – absent* [2, 2, 2]; *respiration – hyperventilating* [1]; *fontanelles – tense* [2]).

The good candidate mentions and excludes *hypoglycaemia, meningitis and electrolyte abnormalities.* (*Correctly calculates severity score 19 = severe HIE).*

### Explaining and planning

The competent candidate uses clear language to explain to the intern what has happened to the baby and uses strategies (*questions, feedback or reverse summarising*) to ensure the intern understands how the diagnosis was made.

The good candidate additionally ensures that the intern is actively involved in managing the baby and learns to apply the HIE scoring system to determine severity and prognosis.

### Management

The competent candidate provides feedback to the intern (what went well, not well and how can there be improvement in the future). Checks that the intern understands and has learnt from the experience.

The good candidate additionally refers the intern to resources for further exploration of the topic.

#### Role play – Instructions for the intern

You are an intern who was called to the nursery to see an infant about whom the nurse was concerned. As soon as you are done with taking the collateral history from the mother, the infant starts to have seizures.

You have minimal experience with neonates and decide to call your consultant to help you. You are not sure what you should do to control the seizures or to manage the baby further.

*Appearance and behaviour*: You are nervous, shocked and very worried.

When your consultant arrives, you answer his questions relating to the history:

- A child born 3 days ago – normal vaginal delivery.
- Apgar score 5/10 and 6/10.
- Prolonged and difficult labour.
- Mother primigravida – 18 years old.
- The child required resuscitation at delivery.
- The mother was booked and was at term.
- No complications during pregnancy.

Familiarise yourself with the patient notes; you can hand them to the candidate if s/he asks for them.

Patient’s notes:

- A 3-day-old neonate, weighing 3 kg, was born to a primigravida who is 18 years old. There were no complications during pregnancy.
- Delivery mode – normal vaginal delivery (NVD), prolonged 2nd stage of labour.
- Apgar scores 5/10 at 1 min and 6/10 at 5 min.
- The midwife’s notes stated that the child needed cardiopulmonary resuscitation (CPR) and bagging at birth.
- Currently, the child is having generalised tonic-clonic seizures.
- She has an IV line and is placed in Servo-Crip (infant warmer) with a temperature set at 36.5 °C.
- Neonatalyte (neonatal maintenance solution) is running at 7 mL/h.
- Before the seizures, the neonate was noted to be stuporous and flaccid with absent reflexes, drooling saliva and poor swallowing.
- She had an absent Moro, grasp and sucking reflexes with a tense fontanelle.

**Further reading**

- Steinberg H. Chapter 33: How to resuscitate a newborn. In: Mash B, Brits H, Naidoo M, Ras T, editors. SA family practice manual. Cape Town: Van Schaik, 2023; p. 136–141.
- Douglas-Escobar M, Weiss MD. Hypoxic-ischemic encephalopathy: a review for the clinician. JAMA Pediatr. 2015;169(4):397–403. https://doi.org/10.1001/jamapediatrics.2014.3269
- Coetzee M. Hypoxic-ischaemic encephalopathy: Identifying newborns who will benefit from therapeutic hypothermia in developing countries. S Afr J Child Health. 2018;12(4):175–180.https://doi.org/10.7196%2FSAJCH.2018.v12i4.1539

## Data Availability

Data sharing is not applicable to this article as no new data were created or analysed in this study.

